# No Impairment in Bone Turnover or Executive Functions in Well-Treated Preschoolers with Phenylketonuria—A Pilot Study

**DOI:** 10.3390/nu16132072

**Published:** 2024-06-28

**Authors:** Beatrice Hanusch, Michael Falkenstein, Stefan Volkenstein, Stefan Dazert, Thomas Lücke, Kathrin Sinningen

**Affiliations:** 1 Research Department of Child Nutrition, University Hospital of Pediatrics and Adolescent Medicine, St. Josef-Hospital, Ruhr-University Bochum, 44791 Bochum, Germany; 2 ALA Institute, 44805 Bochum, Germany; 3 Department of Otorhinolaryngology, Head and Neck Surgery, Johannes Wesling Klinikum Minden, Ruhr-University Bochum, 32429 Minden, Germany; 4 Department of Otorhinolaryngology, Head and Neck Surgery, St. Elisabeth-Hospital Bochum, Ruhr-University Bochum, 44787 Bochum, Germany

**Keywords:** preschool children, bone turnover, phenylketonuria, executive functioning, cognition, calciotropic hormones

## Abstract

Patients with phenylketonuria (PKU) present signs of impaired executive functioning and bone health in adolescence and adulthood, depending in part on the success of therapy in childhood. Therefore, nine children with well-treated PKU (4–7 years old, 22.2% ♀, seven with a full set of data, two included into partial analysis) and 18 age-, gender- and season-matched controls were analyzed for differences in executive functioning and bone parameters in plasma. Plasma was analyzed with commercially available kits. Cognitive performance in tonic alertness, visuo-spatial working memory, inhibitory control and task switching was assessed by a task battery presented on a touch screen. Regarding cognition, only the performance in incongruent conditions in inhibitory control was significantly better in children with PKU than in controls. No further differences in cognitive tests were detected. Furthermore, no significant difference in the bone turnover markers osteocalcin, undercarboxylated osteocalcin and CTX were detected between children with PKU and controls, while children with PKU had a significantly higher vitamin D concentration (69.44 ± 12.83 nmol/L vs. 41.87 ± 15.99 nmol/L, *p* < 0.001) and trended towards lower parathyroid hormone concentrations than controls (48.27 ± 15.16 pg/mL vs. 70.61 ± 30.53 pg/mL, *p* = 0.066). In this small group of well-treated preschoolers with PKU, no impairments in cognitive performance and bone turnover were observed, while vitamin D supplementation of amino acid supplements seems to be sufficient to achieve good vitamin D status.

## 1. Introduction

Phenylketonuria (PKU; OMIM #261600) is a congenital defect of the Phenylalanine (Phe) metabolism with an incidence of 19.3/100,000 newborns in 2020 in Germany [1,2]. Variants in the gene encoding phenylalanine hydroxylases lead to reduced activity of the enzyme, subsequently causing the accumulation of Phe and its metabolites in the blood and brain [1]. Classical symptoms of PKU include irreversible intellectual disability, motor deficits and developmental problems [1]. Early diagnosis and start of treatment, which entails the reduction of Phe intake from natural protein and the supplementation of amino acids, can prevent the development of most symptoms [1,3]. As consumption of natural protein needs to be reduced up until the point of the Phe blood concentrations meeting the target range, overall protein and caloric restriction is not sufficient for normal growth [3]. Therefore, foods naturally higher in protein, such as baked goods and pastas, are available as low-protein substitutes to ensure an adequate energy supply, while protein requirement is covered by Phe-free amino acid mixtures with added vitamins (such as vitamin D) and minerals [3]. While a certain amount of Phe is required for healthy development, a higher intake of Phe results in the elevation of blood Phe concentration and should be limited [1,3]. Adults, therefore, tend towards a vegan-like diet, to reduce the intake of natural Phe [4]. Tetrahydrobiopterin (BH_4_) is the co-factor of phenylalanine hydroxylases, as well as various enzymes involved in neurotransmitter synthesis [1,5]. BH_4_ is available as therapeutic agent for patients responding to the treatment, which enables them to increase their intake of natural protein by ≥100% and/or gain biochemical control [1].

The leading goal in the therapy of patients with PKU is the prevention of neurocognitive deficits [1]. Despite the early diagnosis, children and adults with PKU achieve a significantly lower IQ than controls, but within normal range [6,7,8]. Additionally, executive functioning, e.g., working memory, sustained attention, problem solving and strategy was found to be lower in adolescents and adults with PKU, but less so in children in most but not all studies [9,10,11,12]. The effects of BH_4_ usage and dietary control were also studied in relationship with cognitive outcomes in patients with PKU, and lead to inconsistent results [6,9,10,11,12]. A regression analysis of Phe levels early in life and attention in 57 children with PKU between 7 and 14 years of age showed a significant influence of Phe levels between ages 5 and 7 on attention control and reaction speed [13]. Additionally, lesions sustained in the white matter of the brain were observed in patients with PKU of all ages and seem to be negatively affected by Phe concentrations earlier in life [7,8,14,15]. The ability to sustain attention, suppress impulsivity and renew and connect information at preschool age is important for later academic achievements [16]. Accordingly, preschool age might be especially interesting in the cognitive development of children with PKU. We were especially interested in the attention and executive functioning (inhibition, working memory and task switching) of preschoolers, as these are underlying mechanisms for higher cognitive functions [17,18,19,20]. Executive functions correlate with general intelligence but are not a measure for intelligence itself [21]. Inhibition can be tested by the Flanker Task, in which an interfering item surrounding the goal item slows down the reaction time [17]. Corsi Block Tapping Task can be used to measure the renewing and span of the visuo-spatial subsystem [22]. Switching between tasks requires both inhibition and working memory for accurate and speedy processing [17,19].

However, cognitive disturbances are not the only obstacle patients with PKU have to deal with. For instance, a lower bone mineral density (BMD) was described in several studies in children and adults with PKU [23,24,25,26,27], while some found reduced bone quality in some patients with PKU [28,29,30,31,32]. A systematic review and meta-analysis of bone health in patients with PKU found a frequency of BMD Z-Score between −1 and −2.5 in 28–45% of children, and 5–13% showed BMD Z-Scores below −2.5 [27]. In European adults with PKU, mean BMD Z-Scores were significantly lower than in the reference population, but a low BMD Z-Score (≤−2) only occurred in 1.6–5.5% [25]. While low adherence to diet (and therefore a higher intake of Phe than required for low Phe blood concentration) on the one hand is associated with reduced bone quality and higher spontaneous osteoclastogenesis [24,28], dietary supplementation with amino acids on the other hand might negatively affect bone mineralization [23,33,34,35]. Bone accrual in childhood might influence bone health in older age. Even subclinical disturbances during early childhood could have an impact on peak bone mass, with short-term effects being visible in bone turnover markers rather than in changed BMD, which only depicts bone mineral status but not the fluctuation in bone turnover [36,37,38]. On the other hand, single measurements of bone turnover markers may be limited in their ability to reflect current bone status in children, but an analysis of BMD is reliant upon radiation usage and therefore not without risk [36,39]. Bone turnover is the result of bone resorption and bone formation, which are tightly interwoven with each another. To evaluate bone formation, certain markers from collagen synthesis and osteoblast activity can be used, such as osteocalcin (OCN), alkaline phosphatases, or procollagen 1 N-Peptide [40]. Bone resorption can be measured by collagens released from bone matrix and osteoclast activity markers, such as N- or C-terminal cross-linked telopeptide of type-1 collagen (CTX) as well as Tartrate-resistant acid phosphatase type 5b activity [40].

As described, the studied impairments in bone health and the cognition of patients with PKU are manifold and complex. Since early intervention in cognitive development and bone formation could have a positive effect on health in adulthood, the investigation of corresponding parameters in young, well-treated children with PKU is of interest. We hypothesized that even in young, well-treated children with PKU, the first signs of increased bone turnover and deficits in executive functions compared to healthy age-matched controls can be observed.

## 2. Materials and Methods

**Figure 1:**
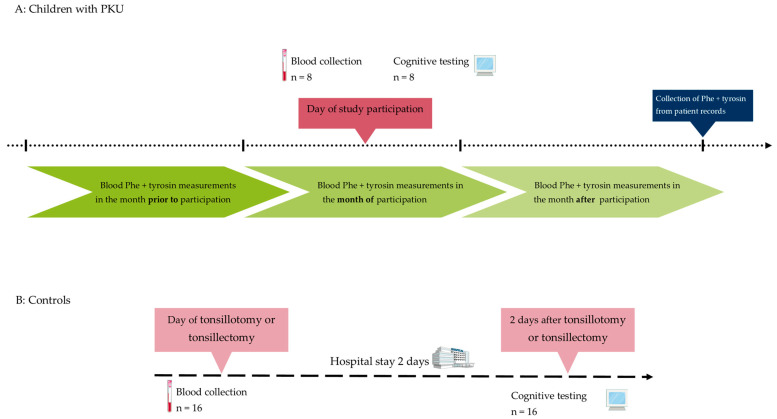
Study protocol for patients with phenylketonuria (**A**) and healthy controls (**B**). On the day of study participation, children with phenylketonuria (PKU) gave fasted blood and took part in the cognitive testing. One child did not give blood, while one child did not participate in cognitive tests. Routine phenylalanine (Phe) and tyrosin blood measurements are carried out regularly multiple times per month in pediatric patients with PKU. These values were collected after study participation for the timeframe of three months surrounding the study participation. In healthy controls, fasted blood was drawn on the day of tonsillotomy or tonsillectomy. Two days after the operation, controls participated in cognitive testing while still in the hospital.

Nine children with PKU (4–7 years old) were recruited to participate during routine check-ups and were characterized by daily Phe tolerance, which is matched to the child’s weight and changes in blood Phe concentration, as well as blood/dried blood filter cards (DBS) Phe concentrations and tyrosine concentrations in the months before, during and after study participation (Figure 1A). Mean Phe and tyrosine concentrations were calculated for these 3 months from a mixture of plasma and DBS results, as DBS usually are collected regularly by the parents. All participating patients were treated with protein restriction and amino acid-supplements; therefore, no child with mild hyperphenylalaninemia was included [1]. Additionally, caregivers of children with PKU gave information on supplement use, other than amino acid supplementation used in treatment. Eighteen age- and gender-matched otherwise healthy preschoolers (4–6 years old) who underwent tonsillotomy or tonsillectomy were recruited to participate as controls, during the same season as the children with PKU. Exclusion criteria were a diagnosed learning disorder or metabolic disease other than PKU, and little or no knowledge of the German language as assessed by clinic staff. In patients with PKU, fasted blood was drawn in the morning on the same day as cognition was tested. For controls, fasted blood was drawn in the morning of the operation. Two days after the operation, digital cognitive testing was conducted at the hospital (Figure 1B). Written informed consent was given by parents or legal guardians of all participants. All participating children gave spoken consent prior to inclusion. The study was approved by the Ethics Committees of Ruhr-University Bochum (No. 17-6311), in accordance with the Declaration of Helsinki.

### 2.1. Biomaterials

EDTA-blood was centrifuged at 3000 rpm at 4 °C for 10 min, and plasma was stored at −80 °C until further analyses. The measurement of the plasma concentration of carboxylated osteocalcin (OCN), undercarboxylated osteocalcin (uOCN), C-terminal telopeptide of type 1 collagen (CTX), parathyroid hormone (PTH), and 25-hydroxy vitamin D (25-OH D) were conducted via commercially available ELISA according to manufacturer’s instructions (OCN and uOCN: EIA kit, Takara, Saint-Germain-en-Laye, France; CTX: Serum Crosslaps^®^, Immunodiagnostic Systems, Boldon Colliery, UK; PTH: Tecan, IBL International GmbH, Hamburg, Germany; 25-OH D: Immunodiagnostic Systems, Boldon Colliery, UK). The 25-OH D was further categorized into vitamin D sufficient (≥50 nmol/L), insufficient (30–50 nmol/L), and deficient (<30 nmol/L), as defined by IOM [41]. Patients with PKU underwent regular blood analysis of Phe and tyrosine. These data for the month before, during and after enrolment in the study were extracted from the patients’ medical records, and mean values were calculated. These were determined by the laboratory for standard care, described as follows: from dried blood filter cards (DBS), discs of 3.0 mm diameter were punched into Eppendorf reaction vials. A total of 20 µL water (LCMS grade) were added to each sample. From each plasma sample, 20 µL EDTA-plasma were transferred into Eppendorf vials. The extraction was carried out with 100 µL Methanol (LCMS grade), containing isotope labeled amino acids (13C, 15N); Sigma-Aldrich, Germany. After vertical shaking at 1000 rpm for 20 min at 20 to 25 °C, all vials were centrifuged at 16,000 RCF for 5 min. The samples were derivatized by 6-aminoquinolyl-N-hydroxysuccinimidyl carbamate in acetonitrile (included in AccQ-Tag™ Ultra Derivatization Kit, Waters, 65760 Eschborn, Germany) with borate buffer. All vials were incubated for 10 min at ambient temperature. The chromatographic separation of partly isobaric compounds was carried out on an ACQUITY UPLC^®^ I-Class System with Cortecs™UPLC^®^, particle size: 1.8 µm; 150 × 2.1 mm (Waters, 65760 Eschborn, Germany) using 0.1% formic acid in ULC water and 0.1% formic acid in acetonitrile as mobile phase. After chromatographic separation, detection was performed using a Xevo^®^ TQS-micro (Waters, Germany) in ESI positive mode quantification with MassLynx™ NT version 4.1 (Waters, Germany).

### 2.2. Cognitive Testing

All participants completed a computer-based cognitive test in a quiet room previously developed in collaboration with the ALA Institute in Bochum, Germany. The tests are based on established pen-and-paper as well as digital tests [42,43,44,45], which were first translated into digital versions for adolescents [46,47,48] and were now adapted to match the abilities of children between 4 and 7 years of age. Tests were presented on a touch-sensitive screen (Hanns-G by Hannspree TH 225 HPB, 5928 PN Venlo, The Netherlands) and were practiced once by the child with the help of study staff. During the actual measurement, each test was explained to the child again by the staff using paper cards and cut-outs of the tests as shown on the screen. All tests are depicted in Figure 1. The overall testing took 20–30 min, with a short break between the practice round and the actual measurement.

### 2.3. Tonic Alertness

**Figure 2:**
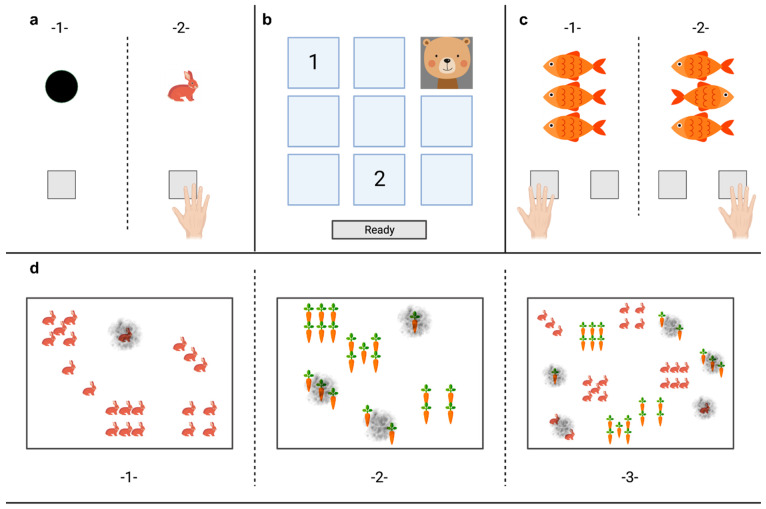
Task battery for executive functioning in children 4–7 years of age. Tonic Alertness (**a**): Children were instructed to tap the grey box as soon as the fixation dot vanished to display a colorful rabbit. Corsi Block Tapping Task (**b**): tested working memory; children were instructed to observe the 3 × 3 matrix on which the animals would appear. They were to remember the order in which the animals appeared on the screen and then repeat this on the 3x3 matrix boxes by tapping on them. Flanker Task (**c**): tested inhibitory control and consisted of congruent (-1-) and incongruent (-2-) stimuli. Children had to tap the box on the side to which the middle fish swam. Switch Task (**d**): The task consisted of three sections. (-1-) rabbits had to be tapped in ascending order, if clicked correctly a grey imprint covered the tapped rabbits; (-2-) carrots needed to be tapped in ascending order; (-3-) Carrots and rabbits had to be tapped alternately (switch) in ascending order (1 carrot–1 rabbit–2 carrots–2 rabbits…).

Tonic Alertness (Figure 2a) was measured by requesting the child to tap on the screen as soon as a black fixation circle on a white screen changed into an illustrated colorful rabbit. The response stimulus interval was 3300 ms (±20%) and maximal accepted reaction time (RT) was set at 1500 ms. The test included 50 items and required the child to stay alert for 3 min. The outcome variables were mean reaction time (RT, ms), the number of missings (no reaction after 1500 ms), and the number of commission errors (reaction during the presence of the fixation circle).

### 2.4. Corsi Block Tapping Task

The Corsi Block Tapping Task (Figure 2b) was used in a digital version and shortened for usage on younger children. A square consisting out of 3 × 3 smaller blue squares was presented on the screen. In these squares, small, illustrated animals appeared for 500 ms with an inter-interval sequence of 1000 ms. Children were asked to remember the location and the order in which these animals appeared. One to four animal-block sequences were displayed and needed to be repeated in the same order. Twelve block sequences had to be repeated with increasing length: 1-, 2-, 3-, and 4-animal-boxes, each three times. As younger children tended to reproduce sequences in inverse order during the testing, additional analysis for correctly tapped boxes, neglecting the correct order, was applied. Therefore, outcome variables were the number of correctly remembered orders and paths, and the number of correct boxes (leaving out the order).

### 2.5. Flanker Task

The Flanker Task (Figure 2c) examines the ability of subjects to suppress dominant responses (i.e., inhibitory control) [49]. Orange fish were used as directional target stimuli and appeared at the center of the screen within confounding variables (flankers vertically arranged). Children were instructed to tap a square at the bottom of the screen indicating in which direction the fish in the middle was swimming (fish swimming right = tap square on the right, fish swimming left = tap square on the left). In the congruent condition, the central fish was flanked by fish swimming in the same direction. In the incongruent condition, the central fish was flanked by fish swimming in the opposite direction. The top and bottom fish were presented first for 100 ms, then the middle fish also appeared on the screen. All three fish were visible to the children for 800 ms. Maximum reaction time was 2000 ms and the response stimulus interval was set as 1000 ms (±20%). The outcome variables were the inverse efficiency (IE) of the congruent and incongruent task (IE = reaction time of trial × (count of all items in trial/count of true reactions in trial)), as well as the difference in IEs (Difference IE = IE incongruent − IE congruent). To avoid implausible results (e.g., due to playing with computer buttons and ignoring the instruction), participants with error rates ≥ 50% in the task were excluded.

### 2.6. Switch Task

The Switch Task (Figure 2d) was developed as a digital version of the Trail Making Task and was adapted to fit the abilities of the young cohort by using rabbits and carrots instead of the usually used letters and numbers. The Switch Task consisted of three sections: the first two sections were non-switch sections, in which one to six rabbits were presented in an irregular order on the screen and should be tapped in an ascending order. The second section presented one to six carrots in an irregular order and children should tap these in an ascending order. The third section was the switching section, in which all rabbits and all carrots were shown and children were asked to “feed the rabbits”: first tap one carrot and then one rabbit; two carrots, two rabbits, and so on. In each section, fields that were tapped correctly were overlaid by a fingerprint. Fields that were tapped incorrectly were not overlaid by a fingerprint. In each section, the maximum time to finish the task was 1.5 min. The outcome variables were the sum of total reaction time (RT) for rabbits (items 2–6) and carrots (items 2–6), and switch costs, i.e., the processing time of the third section minus the sum of the five items of the second section and the five items of the first section (RT_2–6_Switch − (RT_2–6_rabbits + RT_2–6_carrots). Negative switch costs were regarded as implausible and were excluded.

### 2.7. Statistical Analyses

The statistical software package IBM^®^ SPSS^®^ Statistics for Windows, version 29.0 (IBM Corp., Armonk, NY, USA) was used for the statistical analyses. Descriptive data were analyzed by the Chi-squared test or by Fisher’s exact test for groups smaller than five observations. The QQ plots were used to test for normal distribution. Normally distributed data were analyzed using parametric tests (Student’s *t*-test). Non-normally distributed data were analyzed using non-parametric tests (Mann–Whitney U test). Values of *p* < 0.05 were considered significant. Effect sizes for normally distributed data were calculated by using Cohen’s d for groups larger than 20 and Hedges g for groups smaller than 20 observations; Pearson’s r was used for non-normally distributed data. Normally distributed data are presented as mean ± standard deviation (SD), non-normally distributed data as median (25–75th interquartile range). Pearson’s correlation was used to analyze the correlation between normally distributed metric parameters.

## 3. Results

### 3.1. Characterization of Children with PKU and Controls

In total, 27 children participated in the study, and 9 patients with PKU were included, 2 of whom were missing either blood or the cognition test. The 18 controls were age-, gender- and season-matched, 2 for each patient with PKU. Two controls were excluded in blood analysis and another two controls were excluded in cognition analysis.

### 3.2. Characterization of Dietary Control of Patients with PKU

All of the nine participating patients with PKU had mean Phe levels of 79.9–322.7 µmol/L in the months before, during and after study participation. No child exceeded 360 µmol/L, and two children with PKU had mean blood Phe concentrations that stayed below 120 µmol/L (Phe in blood: patient 1: 79.9 µmol/L; patient 2: 101.7 µmol/L, Table 1). In parallel to clinical care with food protocol and counselling whenever needed, six (66.7%) caregivers stated that they calculated Phe ingestion daily, while two (22.2%) said they calculated most of the time, and one caregiver stated that they calculated Phe consumption sometimes. All patients with PKU used amino acid-supplements with tyrosine, and none were treated with BH_4_. Therefore, all participants were affected by PKU and none were affected by mild hyperphenylalaninemia, as defined in European Guidelines [1]. Furthermore, two children with PKU used supplements with docosahexaenoic acid (DocOmega, Vitaflo, Steinbach, Germany), which also contains Vitamin C, sodium, potassium, chloride, calcium and phosphate. No other supplements were used.

### 3.3. Executive Functioning

Overall, 8 patients with PKU and 16 controls were included in the analysis of executive functioning. No significant differences could be found in most subdomains of the executive functioning between the patients with PKU and the controls (Table 2). Only the Flanker Task patients with PKU had a significantly smaller IE in incongruent trials (*p* = 0.030; Table 2), but no significant difference in IE in congruent trials or difference in IEs were detected.

### 3.4. Bone Health

**Figure 3:**
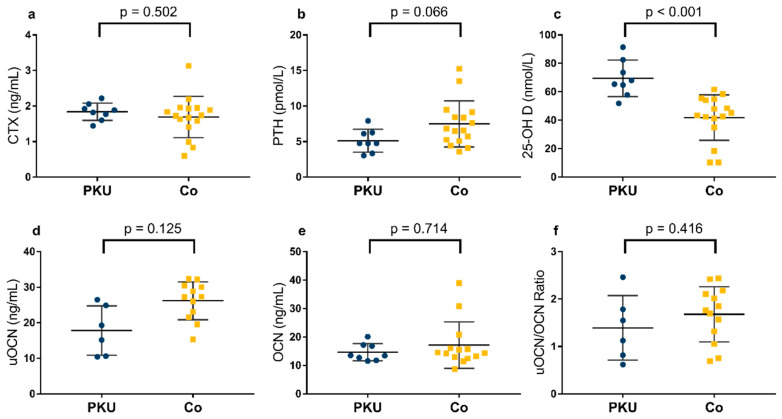
Parameters in plasma relevant for bone health in children with phenylketonuria (PKU) and controls (Co). (**a**) c-terminal telopeptides of type 1 collagen * (CTX; n_PKU _ = _ _8; n_Co _ = _ _16); (**b**) parathyroid hormone * (PTH; n_PKU _ = _ _8; n_Co _ = _ _16); (**c**) 25-hydroxy vitamin D * (25-OH D; n_PKU _ = _ _8; n_Co _ = _ _16); (**d**) undercarboxylated osteocalcin ° (uOCN; n_PKU _ = _ _6; n_Co _ = _ _12); (**e**) carboxylated osteocalcin ° (OCN; n_PKU _ = _ _8; n_Co _ = _ _14); (**f**) ratio of undercarboxylated osteocalcin to carboxylated osteocalcin° (n_PKU _ = _ _6; n_Co _ = _ _13). * *t*-test; ° Mann–Whitney U test. Lines: mean ± standard deviation.

Overall, 8 patients with PKU and 16 controls were included into plasma analysis. No significant differences in CTX (PKU: 1.84 ± 0.24 ng/mL, Co: 1.69 ± 0.58 ng/mL, *p* = 0.502, d = 0.295), OCN (PKU: 13.5 [12.0–17.1] ng/mL, Co: 14.4 [12.9–17.3] ng/mL, *p* = 0.714, r = −0.087), uOCN (PKU: 21.5 [10.6–31.6] ng/mL, Co: 31.4 [24.0–33.8] ng/mL, *p* = 0.235, r = −0.272) and the ratio of uOCN/OCN (PKU: 1.33 [0.77–1.95], Co: 1.76 [1.18–2.14], *p* = 0.416, r = −0.201) were detected between groups (Figure 3). PTH was lower in children with PKU (5.1 ± 1.6 pmol/L) than in controls (7.5 ± 3.2 pmol/L), but this was not significant (*p* = 0.066, d = −0.839, Figure 3). Children with PKU had a significantly higher 25-OH D plasma concentration (69.44 ± 12.83 nmol/L) than controls (41.87 ± 15.99 nmol/L, *p* < 0.001, d = 1.831, Figure 3). Applying cut-offs defined by IOM [41], 25-OH D blood concentration was further categorized into vitamin D sufficient (≥50 nmol/L), insufficient (30–50 nmol/L), and deficient (<30 nmol/L). While no participant with PKU was vitamin D insufficient or deficient, eight (50%) participants in the control group were insufficient and three (18.8%) 25-OH D deficient. Therefore, patients with PKU were significantly more often sufficient in vitamin D than controls (*p* = 0.007). The correlation between PTH and 25-OH D was not significant (r = −0.282, *p* = 0.182).

## 4. Discussion

Older children and adults with PKU showed bone health and executive performance deficits in previous studies [6,7,8,9,10,11,12,13,14,23,24,25,26,27,28,29,30,31,32,33,34,35]. Therefore, an analysis of bone turnover and executive functioning in preschool children with PKU and matched controls was conducted. Neither bone resorption and formation markers, nor most parameters of executive functioning, differed between young children with well-treated PKU and age- and gender-matched controls. Most interestingly, we did find significantly higher vitamin D levels in children with PKU than in controls, even though they were recruited during the same season.

None of the children with PKU exceeded the recommended blood Phe concentration of 360 µmol/L in the months before, during and after participation in the study, and two participants had mean Phe concentrations slightly below 120 µmol/L [1]. As the American College of Medical Genetics and Genomics stated, there is no evidence for the recommendation of blood Phe concentrations of 60–120 µmol/L, but low Phe levels are safe if adequately monitored to avoid prolonged phases of blood Phe concentrations below 30 µmol/L [3]. Therefore, all participants with PKU had good compliance and all consumed amino acid supplements.

The prevention of neurocognitive deficits in patients with PKU is the leading treatment goal in the care of the metabolic disease [1]. We observed no difference in performance in the Corsi Block Tapping Task, Tonic Alertness Task and Switch Task in well-treated preschool children with PKU and healthy controls. As Townsend and Ashby suggested in 1978, and Bruyer and Brysnaert discussed in 2011, speed and accuracy in some cognitive testing can influence each other in a speed–accuracy trade-off [50]. Therefore, we used the inverse efficiency (IE) as discussed to evaluate Flanker Task performance [50]. The IE penalizes faster RT if this comes with a higher number of false reactions. Therefore, higher IE indicates poorer performance in the Flanker Task [51]. We observed significantly better performance in processing the incongruent stimuli in children with PKU vs. controls. Matching our results, Paermentier et al. observed no significant difference in the spatial working memory of pre-school children with hyperphenylalaninemia and controls, but better performance in inhibitory control in pre-school children with hyperphenylalaninemia. As they included children with moderate hyperphenylalaninemia and phenylketonuria, they were able to compare these two groups as well, and found lower performance in inhibitory control and spatial working memory in children with PKU compared to children with moderate hyperphenylalaninemia (none treated with protein restriction) [52]. As all participants in our study had PKU, we were not able to compare participants with classical PKU and hyperphenylalaninemia in our cohort. In the Netherlands, children with PKU were recruited to participate in a study analyzing executive functioning and its associations to Phe blood concentrations in 1997–1998 [13,53,54,55]. Comparable to our results, children with Phe blood levels below 360 µmol/L did not differ from healthy controls in inhibition and attention flexibility [53,54], whereas in the baseline speed task (comparable to the attention testing we used), all patients with PKU had longer RTs than age-matched controls [13]. In 2012–2015, all patients were re-invited to participate. In this adult group of early-treated patients, a significant correlation of Phe levels during early childhood (0–12 years), as well as during adolescents (13–17 years), and executive functioning was observed. For inhibitory control (Flanker Task), a greater increase in blood Phe levels from childhood to adulthood correlated with poorer performance in the task, while cognitive flexibility was correlated with Phe levels in early childhood [55]. On the contrary, another study revealed a trend towards higher error rates in NoGo trials of the Go-NoGo test (reduced inhibitory control) in young adult female patients with PKU compared to healthy age-matched controls. These results did not correlate with the average Phe blood concentration during the first six years of the lives of these patients, but came with a relative increase in brain activity in the right middle frontal gyrus [56]. Another study showed that 29 out of 30 patients with PKU had lesions, especially in the parietal and occipital lobes [57]. Additionally, working memory, cognitive flexibility, sustained attention and processing speed were measured in these patients with PKU and compared to controls, describing significantly lower performance in the patients. Neither historical nor current metabolic control in those patients was associated with performance in the cognitive testing and the white matter lesions, after correcting for multiple comparisons [57]. In children with PKU, Hood et al. observed correlations of mean diffusivity of two white matter brain regions with lifetime exposure to Phe and with performance in executive functioning testing [58]. Similarly, 86 children (8–17 years old) showed significant improvement in parent-reported executive functioning after blood Phe concentration was reduced due to treatment with BH_4_ [59]. We observed no difference in the performance of visuo-spatial working memory, task switching or tonic alertness, but better performance in the inhibition of incongruent stimuli in children with PKU compared to healthy controls. Since a significant difference was neither observed in processing the congruent stimuli nor in the difference of IE, further analysis of inhibitory control in young children with PKU is needed. As children included into the present study were well treated, with no patient exceeding the upper recommendation of Phe level for the age group, it might be possible for executive functioning to still be unaffected in these young children, which in turn does not exclude the possibility for the occurrence of damages in the brain and performance issues later in life. Because the children included into our pilot study had lower blood Phe concentrations than those in the study of Paermentier et al., their performance in the inhibition task seems comparable to the children with moderate hyperphenylalaninemia [52].

In the IDEFICS study, children between 3 and 15 years of age from eight European countries had a mean 25-OH D serum concentration of 45.2 (±16.7) nmol/L, with 37% of children being classified as vitamin D sufficient and significant differences between countries [60]. Controls in our sample reached vitamin D sufficiency in 31.3% of cases, consistent with the IDEFICS observations. The 560 German children included in the IDEFICS study had a mean 25-OH D serum concentration of 36.1 (±15.1) nmol/L [60]. In 2007, data from the German KiGGS cohort study on 25-OH D in the serum of 10,115 children living in Germany were published. In the group of 3–6 year olds, median serum 25-OH D was 44.1 nmol/L (p5–p95: 15.0–95.8 nmol/L) [61], which corresponds to the controls in our study. On the contrary, median serum 25-OH D in 1–2 year olds in KiGGS was the highest of all age groups at 61.9 nmol/L (p5–p95: 19.4–115.0 nmol/L) [61], matching the 25-OH D concentrations of patients with PKU in our pilot study. It is recommended for infants in Germany to be given vitamin D supplementation until their second summer of life (e.g., up until about 1.5 years of age) [62]. Kunz et al. found clear differences in the vitamin D status in German children according to season [63]. In 967 participants between 0 and 17 years of age, vitamin D sufficiency in 2013–2014 was observed to be 56%/39.7% during summer/autumn, while only 29.2% and 28.1% reached sufficiency in winter and spring [63]. Overall, children in our pilot study were mostly recruited during summer and autumn, when UV exposure and time spent outside tend to be higher. Therefore, the results presented here fit into the current literature for healthy children living in Germany. Accordingly, the good vitamin D status of the children recruited with PKU is interesting. As vitamin D is either taken in by food sources or dietary supplements, or is produced by the skin as it is exposed to ultraviolet (UV) radiation [64], we matched patients and controls not only for age and gender, but also for recruitment season. Thus, differences in sun exposure are less likely and differences in vitamin D from foods or supplements might account for the higher vitamin D concentrations in patients. The recommended vitamin D supplementation in infants explains the higher 25-OH D concentrations in younger children, as described in KiGGS [61] and by Geserick et al. [65]. All patients with PKU consumed amino acid supplements containing 2.5–5 µg vitamin D/portion (xPHE energy, xPHE enjoy, and xPHE hello, metaX, Friedberg, Germany; PKU gel and PKU cooler, Vitaflo, Steinbach, Germany; Glytactin, Cambrook, Dali, Nicosia, Cyprus). The parents or guardians stated that the included children with PKU consumed about 2.5 to 3 portions of amino acid supplements and no further Vitamin D supplement each day, resulting in a vitamin D intake of 6.25–9 µg per day from these amino acid supplements. At the same time, healthy children between 6 and 8 years of age in Germany consume on average 1.8 µg of vitamin D from food [66]. Therefore, the higher vitamin D plasma concentration in PKU patients probably results from intake of amino acid supplements. In their study on bone mineral density in patients (aged 8–20 years) Geiger et al. also did not find a patient with PKU with vitamin D deficiency [30]. In contrast, Demirads et al. found that of 60 patients with PKU (aged 1–39 years), 12% were not vitamin D sufficient, with 4 using Phe-free amino acid mixtures (1 without added vitamin D) and 3 who did not adhere to a restrictive diet [32].

Next to a significantly higher 25-OH D plasma concentration in children with PKU, we found lower PTH plasma concentrations in these children compared to controls, which did not reach significance. Several studies described a negative non-linear relationship between 25-OH D and PTH in children, with a plateau at about 75 nmol/L 25-OH D [67,68,69]. Therefore, the trend for lower PTH levels in our patients with PKU can be a result of higher 25-OH D levels, but the lack of significance might be a result of the small group of analyzed children. Additionally, the lack of a significant correlation between PTH and 25-OH D could be a result of the non-linearity of the relationship between the two hormones, with a flattening of the curve before the plateau [67]. As no data on PTH from the IDEFICS observations or the KiGGS [70] on PTH in children exist, comparing controls in our study to healthy children living in Germany was not feasible. Sahin et al. [67] and Vissing Landgrebe et al. [71] measured PTH in children. Compared to both studies [67,71], PTH in our controls was higher than expected, but 25-OHD was lower. The regression analysis conducted by Vissing Landgrebe et al. resulted in an increase of 1 pmol/L of PTH for each 4.6 nmol/L decrease of vitamin D in serum [71]. As the controls in our study were younger and had vitamin D concentrations of 21 nmol/L lower than the participants in Vissing Landgrebe et al.’s study, higher PTH concentration might result from these differences [71]. Higher 25-OHD concentrations were also observed in Sahin et al.’s study, with children of the same age as in our study [67]. As for patients with PKU, PTH concentration in plasma was also slightly higher than in the studies referred to, but still within, their standard deviation [67,71].

Bone formation and resorption are continuous processes that do not only occur during growth or bone mass accrual. In 395 7-year-old children from Portugal, no correlation of total body bone mineral content and bone formation and resorption parameters was observed after age, body size, and season were considered. Single measurements of these markers may be limited in their ability to reflect current bone status in children [39]. As a marker of bone resorption, CTX was analyzed in this pilot study. In children, CTX rises from birth to puberty, with a median CTX concentration of about 1.5 ng/mL at 6 years and a p95 of about 2.5 ng/mL [65]. According to these reference values, the bone resorption in children included into our pilot study was within the normal range. For OCN, a bone formation marker, Geserick et al. observed high values during the first year of life [65], which subsequently fell to a nadir between 3–4 years of age and then rose again. Previously, Johansen et al., van Summeren et al. and Popko et al. found uOCN and OCN serum concentrations comparable with those we found in plasma [72,73,74], whereas Tubic et al. found uOCN at a mean concentration of 7 ng/mL and OCN at a concentration of 75.6 ng/mL, and Geserick et al. described median OCN at 3–4 years of age of 74.5 ng/mL in boys and 78.1 ng/mL for girls [65,75]. While van Summeren et al. and Popko et al. used the same ELISA assays we used here [73,74], Johansen et al. used radio-immunoassay [72], Tubic et al. and Geserick et al. used an electrochemiluminescence immunoassay on Cobas instruments, which explains the contradicting results [65,75]. To our knowledge, this is the first study that analyzed uOCN in well-treated young children with PKU, which was described to have hormonal effects in mice acting on glucose sensitivity and neurotransmitter production [76].

A limiting factor of this study was the small number of patients included due to its design as a pilot study. In addition, only eight patients with PKU were able to give blood, and one child with PKU was not able to participate in cognitive testing. As the drawing of blood in healthy children is problematic, children undergoing a small surgical procedure were used as controls. To secure no influence of sedation on the test results of cognitive battery, children were tested two days after the surgery. It cannot be excluded that pain or malaise could have influenced the performance of controls. Furthermore, the small amount of blood collected did not allow for a thorough analysis of bone-related parameters. Children with PKU did not only give blood for study purposes, but also for routine check-up, to minimize the times children had to be punctured. Moreover, the age range of 4 to 7 years is a timeframe during childhood when growth spurts occur [77] and cognition is developing rapidly [78]. Because growth spurts are difficult to predict [77], we cannot rule out that the children included in this analysis were undergoing a spurt at the time of blood collection, which in turn might have influenced the parameters of bone formation and resorption. Parameters such as height or weight would have been helpful in interpreting the results, but were regrettably not collected. As cognitive functions are rapidly developing during early childhood [78], the tests might have been too difficult for younger children and/or too easy for older children. We tried to eradicate this by matching patients with controls of the same age, but since calendar age does not necessarily reflect brain maturity [78], results have to be interpreted with caution. Additionally, although based on well-established pen-and-paper tests, the battery used for cognitive testing was not validated; however, this is in part compensated by the direct comparison of patients with controls. Vitamin D supplementation in controls was not documented. As 25-OHD concentration in these children was comparable to children in the German KiGGS cohort study [61], it can be expected that the children did not take any vitamin D supplementation. Additionally, the usual diet in children with PKU and early-life management was not analyzed, but could have influenced data on cognition and bone formation and resorption. This data should be included in future studies. As this study was a pilot study, we are interested in further investigating bone health, cognition and the relationship between these parameters with habitual diet, physical activity and Phe concentration in older individuals. In particular, we would like to include older individuals to study a broader spectrum of parameters, as this allows for larger volumes of blood to be obtained and reduces the impact of developmental spurts.

## 5. Conclusions

In conclusion, we did not find an early indication for preventable comorbidities in well-treated young children with PKU compared to healthy age-, gender- and season-matched controls. No child with PKU included was vitamin D insufficient or deficient, with no effect on bone formation or resorption markers. Additionally, no difference in executive functioning was observed in the children with PKU compared to the controls. Nevertheless, many studies describe impairments in bone turnover and cognitive performance in older children with PKU. Continuous treatment and regular check-ups should be performed to prevent the development of these impairments and to ensure early intervention.

## Figures and Tables

**Table 1:** Characterization of patients with phenylketonuria (PKU) and controls (Co).

Parameter	PKU	Co	*p*
n	9	18	
Age (years)	5.4 ± 1.2	5.1 ± 0.8	0.464 ^a^
Female n (%)	2 (22.2%)	4 (22.2%)	1.000
Winter/spring n (%)	2 (22.2%)	5 (27.8%)	1.000
Daily Phe tolerance (mg/d)	291.1 ± 43.4	n.a.	-
Mean Phe in blood * (µmol/L)	203.4 ± 82.9	n.a.	-
Mean Tyr in blood * (µmol/L)	80.0 ± 34.2	n.a.	-
Supplementation with AAS n (%)	9 (100%)	n.a.	-

*mean values of regular routine measurements from the patients record in the month of the study participation, prior to and after inclusion, ^a^ Cohen’s d = 0.304, AAS—amino acid-supplements, n.a.—not applicable, Phe—phenylalanine, Tyr—tyrosine.

**Table 2:** Executive functions in patients with phenylketonuria (PKU) and controls (Co).

Parameter of Executive Functioning	PKU	Co	*p*	Effect Size
Tonic Alertness °				
Commission error (n)	6.00 ± 5.60	8.27 ± 7.44	0.461	−0.329 ^a^
Missing (n)	2.5 [0.3–4.8]	5.0 [2.0–9.0]	0.213	−0.264 ^c^
Average RT correct (ms)	701.2 [621.3–814.2]	738.8 [656.9–905.0]	0.506	−0.148 ^c^
Corsi Block Tapping Task ^$^				
Correct order and path (n)	7.63 ± 1.10	7.19 ± 0.60	0.706	0.165 ^a^
Correct boxes (n)	9.13 ± 0.79	9.31 ± 0.49	0.835	−0.092 ^a^
Flanker Task ^~^				
IE congruent (ms)	886.7 ± 226.2	1029.9 ± 161.5	0.139	−0.741 ^b^
**IE incongruent (ms)**	**1036.8 ± 194.9**	**1239.9 ± 157.9**	**0.030**	**−1.135 ^b^**
Difference IE (ms)	150.2 ± 95.6	210.0 ± 130.7	0.337	−0.472 ^b^
Switch Task *^,#^				
Sum RT lblast * (s)	17.8 [10.2–39.1]	17.5 [14.2–27.0]	1.000	0.000 ^c^
Switch costs ^#^ (s)	29.6 [12.2–45.8]	21.2 [16.2–24.8]	0.606	−0.160 ^c^

° n_PKU _ = _ _8, n_Co _ = _ _15. ^$^ n_PKU _ = _ _8, n_Co _ = _ _16. ^~^ n_PKU _ = _ _6, n_Co _ = _ _11. * n_PKU _ = _ _6, n_Co _ = _ _11. ^#^ n_PKU _ = _ _5, n_Co _ = _ _9. ^a^ Cohen’s d, ^b^ Hedges g, ^c^ Pearsons r. IE—inverse efficiency; RT—reaction time; significant results are marked in bold.

## Data Availability

The datasets used and analyzed during the current study are available from the corresponding author on reasonable request.

## Abbreviations

25-OH D	25-hydroxy vitamin D
BH_4_	Tetrahydrobiopterin
BMD	bone mineral density
CTX	C-terminal telopeptide of type 1 collagen
DBS	dried blood filter cards
IE	inverse efficiency
OCN	carboxylated osteocalcin
Phe	phenylalanine
PKU	phenylketonuria
PTH	parathyroid hormone
RT	reaction time
SD	standard deviation
uOCN	undercarboxylated osteocalcin
UV	ultraviolet
WHO	World Health Organization
